# Innovation for Positive Sustainable Legacy From Mega Sports Events: Virtual Reality as a Tool for Social Inclusion Legacy for Paris 2024 Paralympic Games

**DOI:** 10.3389/fspor.2021.625677

**Published:** 2021-04-21

**Authors:** Terri Byers, Emily Jane Hayday, Fred Mason, Phillip Lunga, Daneka Headley

**Affiliations:** ^1^Faculty of Kinesiology, University of New Brunswick Fredericton, Fredericton, NB, Canada; ^2^Institute for Sport Business, Loughborough University London, London, United Kingdom

**Keywords:** legacy, mega sport event, sustainability, innovation, virtual reality, social inclusion

## Abstract

There is significant interest in how sports events and their associated legacies could act as a platform to address global challenges and engender social change. The United Nations (UN) has acknowledged the important role that sport plays in supporting the UN 2030 Agenda for Sustainable Development and the Olympic movement could be argued as central to that objective. Yet critical questions and concerns have been raised about the growing expenditure, viability, long term legacy, and impacts of mega sports events such as the Olympic Games. While much evidence has focused on the challenges of creating legacy for Olympic Games, there is considerably less literature on understanding the Paralympic context. The purpose of this paper is to discuss the role of innovation in creating legacy from MSEs and propose a theoretical and methodological plan for such research. Innovation, a key driver in organizational performance, is suggested as essential to defining, planning for and measuring legacy. We specifically examine the potential of virtual reality (VR) as a technological innovation which can help create a social inclusion legacy in the context of Paris 2024 Olympic/Paralympic Games. A conceptual model is developed, which identifies legacy as a “wicked problem”, and this paper discusses the importance of innovation with regards to legacy, by suggesting a new application for VR technology in the context of legacy related to social inclusion. Information technology is a valuable facilitator of social inclusion for individuals with a disability. We specifically examine the potential of VR as a technological innovation which can help create legacy through influencing unconscious biases (symbolic ableism) toward diversity such as disability, gender, and race.

## Introduction

Theoretically, mega sport events (MSEs) offer a mechanism through which social outcomes and inclusive communities can be created. There is significant interest in how sporting events and their associated legacies could act as a platform to address global challenges and engender social change, as evidenced in the UN 2030 Agenda for Sustainable Development and through the cooperation between the UN and the International Olympic Committee (IOC). The Olympic and Paralympic Games are two of the largest sporting events in the world and the IOC's (2017) sustainability Strategy priorities align with a core number of the UN Sustainable Development Goals (SDGs), culminating in a clear mission to aid social development through sport. However, critical questions and concerns have been raised about the ever-growing expenditure, viability, long-term legacy, and impacts of MSEs, such as the Olympics Games, which has resulted in doubt and discussion around the value of hosting such events (Giulianotti et al., 2015; Flyvbjerg et al., 2016; Zimbalist, 2020). With noticeable downturns in the number of countries bidding to host the Games (Schnitzer and Haizinger, 2019), sustainable development is critical to the long-term viability of the Olympic movement. The focus on sustainability and achievement of legacy outcomes associated with hosting MSEs has never been more important.

Legacy can be positive or negative and although there is still a lack of consensus within the academic field regarding how best to conceptualize, achieve, and evaluate certain forms of legacy, significant levels of research and focus have been placed on mega event legacy (Preuss, 2007, 2015; Leopkey and Parent, 2012; Brittain et al., 2018; Thomson et al., 2019). The uncertainty of the future of sport with regards to COVID-19, especially live mass spectator events, paired with the fragile economic situation and global instability, suggest MSEs should not only reflect and justify their worth and feasibility, but also ensure they are contributing and supporting broader global sustainable development objectives and outcomes. Sustainability can be conceptualized in many ways, in reference to facilities, the environment/climate, economy, resources, or social-cultural context, and Tokyo 2020 (post-poned until 2021) was the first to explicitly ensure alignment with the UN's SDGs across their planning, strategy, and legacy objectives for the Games (Tokyo Organising Committee of the Olympic and Paralympic Games, 2016). Yet, it is important to note that the COVID-19 pandemic has resulted in a global humanitarian, human rights, and socio-economic crisis, thereby stunting the progress of the UN's Sustainable Development Goals 2030 (Sustainable Development Goals Report, 2020).

While much evidence has focused on the challenges of creating legacy for the Olympic Games (Brittain et al., 2018), there is considerably less literature on understanding the Paralympic legacy context (Misener et al., 2013; Darcy, 2016; Pappous and Brown, 2018). Exceptions include Brittain and Beacom's (2016) work demonstrating that the UK government's planned legacy benefits of hosting the 2012 Olympic and Paralympic Games were contrary to the findings of Disabled People's Organizations (DPOs). The DPOs interviewed over 1,000 people living with a disability and noted unchanged negative attitudes and experience of aggravated hostiles in some cases (Scope, 2013). Yet, some positive impacts have been noted with Coates and Vickerman (2016) arguing that the inspirational impact of Paralympians has positively influenced young people's attitudes toward disability. Given that social inclusion is a major priority for the Paris 2024 Olympic/Paralympic Games, it is urgent to consider innovative solutions that could be used to achieve social legacies for persons with a disability, given the negative perceptions of disability recorded in the literature (Brittain and Beacom, 2016; Brown and Pappous, 2018; Brittain et al., 2020).

Innovation has been explored in a variety of contexts with some focus on sport organizations (Füller et al., 2007; Hoeber et al., 2015; Wemmer et al., 2016). Literature shows a focus on how imperative innovation is toward gaining and maintaining a competitive edge in commercial and voluntary sport organizations (Hoeber et al., 2015; Tjønndal, 2016). Yet, there is a lack of engagement with the concept of innovation, within the context of legacy. To date, legacy production and advances in understanding the legacy of MSEs have been built upon rational decision making, planning, and strategic leveraging (Preuss, 2007; Chalip, 2017; Girginov et al., 2017). However, due to the complex and “wicked” nature of legacy production (Byers et al., 2020), innovation may provide a valuable mechanism through which certain legacies can be achieved. The purpose of this paper is to provide a conceptual analysis of the role of innovation in creating a sustainable social inclusion legacy for persons with a disability through MSEs and propose a theoretical and methodological plan for such research.

We specifically examine the potential of virtual reality (VR) as a technological innovation, which can help create a social inclusion legacy for Paris 2024, by influencing unconscious biases toward diversity such as disability, gender, and race. In this paper, we see disability as a form of diversity, which includes personal, cultural, and institutional differences (Patrick and Kumar, 2012). Although this study is specifically focusing on disability, application is possible across other diversity-based characteristics. We propose that, as a technological innovation, VR delivered through diversity training in sport organizations or used as a recruitment tool to engage the participation of disabled persons, could increase empathy toward persons with a disability, decrease social ableism (of and in persons with a disability), and therefore foster greater social inclusion in sport organizations. VR has been proven as a powerful mechanism used to tackle discrimination, promote diversity, and encourage attitudinal change (Beadle and Santy, 2008; Bielen et al., 2018; Lopez et al., 2019) but has yet to be applied in the context of sport, as an innovative approach to achieve a social inclusion legacy.

From a policy context, the European Union identified technological development, specifically augmented and virtual reality, as a key priority (EU, 2017), as well as investment in MSEs. This paper offers an innovative solution to how these priorities may be realized through MSEs legacy and the Paris 2024 Games. The challenges of using VR as a tool in sport organizations are explored and thoughts for future research in this area are provided. This paper is structured in three parts. First, we provide some context for the Paris 2024 Olympic/Paralympic Games and how this connects to EU priorities of diversity, inclusion, and innovation, and to the broader UN mandate and SDGs, which aim to create a sustainable future for all. Next, literature on legacy is reviewed with key gaps identified indicating that perceptions and attitudes to disability are key barriers challenging the production of legacy, including Paris 2024. Then we relate this review to how sport organizations have approached legacy, suggesting a lack of innovation related to legacy production. Building on the conceptual work of Byers et al. (2020), a practical application to a specific context is proposed and thoughts for research implications are provided. Critically, the need for more conceptual work focusing on social inclusion has been acknowledged (United Nations, 2016), which supports the need for such research into the complexities of social inclusion and its production.

## Legacy, Disability Sport, and Innovation: Toward Social Inclusion

### Context

In 2024, France will host the Olympic and Paralympic Games, having spent more than 8 years planning for the event and its legacy. To this end, the Organizing Committee of the Olympic Games (COJO) has drawn up the “Generation 2024” program with the aim of setting priority lines of action in order to make the event a lever for building a more inclusive society. Through this program, the aim is to produce a sustainable Olympic and Paralympic legacy for all French citizens, including disabled persons (Paris, 2020). In this context, the Olympic and Paralympic event is seen as a way to “shift the perception” of disabled people from challenged toward ability/achievement and increase social inclusion. Critically, Paris 2024 has designed their legacy and sustainability plan to ensure it is fully aligned with the UN's SDGs, and this strategy is being supported by stakeholders such as UNICEF France (Olympic.org, 2020). One of Paris 2024's core legacy pillars is to create a more inclusive society and here lies the opportunity to consider how innovation could be key to achieving such an ambitious objective. Through both the plans outlined by Tokyo and Paris, there is a pattern emerging through which the Olympic and Paralympic Movement (and sport more broadly) are being used as mechanisms to support UN priorities and work toward a sustainable, inclusive future for all.

Diversity and inclusion are frequently discussed as critical social imperatives, forming the foundation of multiple SDG's and have become a consideration within multiple facets of society (Roberson, 2006; Forde et al., 2015; Inoue, 2019; Kang and Kaplan, 2019; Schuelka et al., 2019; Kim, 2020). Social inclusion is defined as “the process of improving the terms of participation in society, particularly for people who are disadvantaged, through enhancing opportunities, access to resources, voice, and respect for rights” (United Nations, 2016, p. 17). Through directives, policies, and quotas, the acknowledgment that diversity should be supported and encouraged through inclusive practices has never been more evident (Zhao and Zhang, 2018; Martínez-Ariño et al., 2019; Legislation.gov.uk, 2020; Rankin, 2020; UNESCO, 2020). Yet, there are still clear examples of inequalities, exclusion, and injustice, with people with disabilities being one of the groups often affected (Jaeger and Bowman, 2005; McConkey et al., 2013; Shandra, 2018; van Trigt, 2019). Inequality and exclusionary practices present barriers limiting the social inclusion of disabled people and are underpinned by negative attitudes known as ableism (Brittain et al., 2020).

Considering the expectation of MSE's to leave a lasting impact or legacy, it is understandable that ambitious plans, such as Paris 2024's focus on inclusion (a Games for all people), are outlined. Reflecting on Paris 2024 and the European context, diversity and inclusion have been identified as key areas for consideration, as evidenced in the EU (2017). Whether looking through a European or global lens, innovation has been noted as a critical component to achieving the SDG's. As these goals aim to tackle global challenges that have been prolific in our societies for generations, Whelan (2020), lead of the UN's Global Compact team, has noted that breakthrough innovations driven by new business models, mindsets, and disruptive technologies are required. Specifically, in line with Paris 2024's social inclusion agenda, if we focus on SDG 10, which aims to reduce inequalities (and specifically note #Envision2030—Imagine the world in 2030, fully inclusive of persons with disabilities—United Nations, 2020), new innovative approaches should be considered to tackle this complex and “wicked” problem.

Disability sport has been studied from multiple disciplinary perspectives, revealing that the concept is complex, multidimensional, and contestable to both define and operationalize (Misener and Darcy, 2014). Medical views of disability focus on categorization of physical or intellectual impairments (World Health Organization, 2001). From this view, common barriers to sport participation for persons with a disability can include lack of awareness of how to include diverse populations, limited programs and accessible facilities, transportation, and access to resources/information (DePauw and Gavron, 2005). Given the nature of an impairment, practical steps in service delivery and program design can be taken to accommodate for the disability. This is seen in the “inclusion spectrum” approach to increasing social inclusion of persons with a disability to sport (Kung and Taylor, 2014), where various degrees of integration in mainstream sport/programs is offered by a sport organization. This includes a spectrum of programing from fully integrated without adaptation of the event/program to discrete activities where participation is with peers with similar disabilities. However, several authors suggest that a reconceptualization of inclusion in sports for people with disabilities is needed (Promis et al., 2001; Grandisson et al., 2012).

Disability sports has a history of fragmentation and unequal access, in part due to perceptions of people with disabilities. Sports for persons with disabilities has tended to be organized on a disability-by-disability basis, resulting in clear forms of separation, as for example, separate sports organizations exist for individual forms of disability such as wheelchair users or athletes with cerebral palsy (Doll-Tepper, 1999; Bailey, 2008). This has resulted in delivery challenges, especially for multi-sports events, with tensions between groups over organization and control, as well as access issues for individuals who have a disability which does not fit easily into a category (Mason, 2002; Buttimer and Tierney, 2005; Brittain, 2010). There have been challenges in getting sports media to perceive (Solves et al., 2019) and cover disability sport as elite sport (Howe, 2008; Mason et al., 2010; Mason, 2013), and athletes whose physical bodies do not fit into perceptions of “athlete” because of their disability tend to not receive coverage (Bruce, 2014). Related to all of this, people with physical disabilities tend to have more opportunities, with more organizational support, compared to those with intellectual or invisible disabilities (Moran and Block, 2010). These studies highlight that for individuals with a disability, there is still limited social inclusion and discriminatory practices within the sports industry. Could innovation be a solution?

Innovation across the EU is considered essential to European competitiveness in the global economy. Innovation in small and medium-sized enterprises is particularly important to building entrepreneurship and industry growth across the EU. One technological innovation that is experiencing exponential growth in the EU is virtual reality (VR), with spending on these products and services expected to grow by 72% worldwide by 2022 (CBI, 2020). Furthermore, with a COVID-19 socially distanced society, the potential for VR applications is significant as an alternative mechanism to promote and actually experience diversity in a safe environment. Virtual experiences often produce greater attitudinal change and understanding than face to face interactions (Hudlicka, 2013). Therefore, as a technological innovation, VR may not only provide a dynamic and novel approach to produce a social inclusion legacy, but also offer a suitable and safe mechanism to support this social imperative through legacy in a time of significant uncertainty.

### Challenges: Existing Literature on Legacy

In 1999, the IOC decided to create a version of the United Nations' (UN) Agenda 21 for Sustainable Development which they called the Olympic Movement's Agenda 21 for Sustainable Development. To justify the taxpayer-funded high cost of hosting MSEs, organizing committees, with the support of influential media, would pitch for the economic dividends a host city, region, or country would realize from the event (Cornelissen and Swart, 2006; Swart and Bob, 2007). The argument for hosting the Paralympic Games has been to create a platform where resources are used to develop accessible infrastructure and transportation networks (Darcy, 2001; Shuhan and LeClair, 2011). These economic legacy guidelines, together with planning for social changes to counter negative public perceptions of disability whilst empowering people with a disability, are an integral part of the IOC bid assessment (Misener et al., 2013). However, as mentioned earlier, none of the studies have adequately addressed or measured IOC's Agenda 21 goals (Darcy, 2003; Weed and Dowse, 2009).

Legacy, prior to the Sydney 2000 Olympics, had been based on event owners/organizing committee accounts rather than independent research (Preuss, 2007; Misener et al., 2013). With the Olympic Movement falling into the traps of commercialization, and the advent of the politicization of hosting MSEs, it has become important that these institutions focus on perceived positive outcomes (Cashman, 2006; Girginov and Hills, 2008). Critically, investigations concerning legacy outcomes have been inconclusive (McCartney et al., 2010; Mahtani et al., 2013; Brittain et al., 2018) and this could be due to failure to leverage hosting benefits by organizing committees (Annear et al., 2019), a disparity between political rhetoric and measurable outcomes (Brittain et al., 2018), ineffective research designs (Mahtani et al., 2013), and/or sampling errors (McCartney et al., 2010). Legacy production from MSEs has been a challenge for nations with conflicting evidence of long-term impacts (Brittain et al., 2018). Several scholars have developed theories to anticipate and explain MSE legacy effects; according to Annear et al. (2019), the most commonly mentioned are the demonstration effect, festival effect, and social ecological model. Conducting a systematic review using the demonstration theory, Annear et al. (2019) revealed that using the Paralympics to inspire other physically disabled people did not produce the intended long-term legacy. On the other hand, the festival effect which could be an outcome of the extensive media coverage during the Games, left inactive people positive about the MSEs, but the long-term impact of that positivity is unclear (Carter and Lorenc, 2015; Pappous and Hayday, 2016). The social ecological model theory was more evident in the Tokyo 1964 Olympic Games, whereby the generation that experienced the MSE participated in sport more frequently than other generations who did not (Aizawa et al., 2018).

Dickson et al. (2011) suggested that there is a dearth in Paralympic legacy research, and focus to date has been directed toward identifying barriers to attitudinal change toward disabled individuals and mechanisms to increase social inclusion (Ferez et al., 2020). Specifically, the Paralympic Games are organized in parallel with the Olympic Games, in part to foster legacies of inclusion for host nations, giving athletes with physical disabilities the opportunity to inspire other people living with disabilities (Gold and Gold, 2007). According to Misener et al. (2013), the legacy objective of the London 2012 Olympic/Paralympic Games to create a social legacy for disabled athletes, volunteers (including increased empathy and positive attitudes toward disability) was unsuccessful. Pappous and Brown (2018, p. 651) note “although the London 2012 Paralympic Games were considered a success, doubts remain on the impact it has had on the lives of disabled people in the UK.” Other scholars have also questioned the achievement of Paralympic legacy objectives (Ahmed, 2013; Bush et al., 2013), with few studies identifying positive outcomes with regards to visibility for people with disabilities and inspiration for children with disabilities (Coates and Vickerman, 2016; de Souza and Brittain, 2020). Coverage of Paralympians has also often been shown to be unconsciously biased to promote weakness, sympathy, and negative connotations of disability rather than focus on the strength, ability, and positive achievements of persons with disability (Pappous et al., 2011), again thwarting legacy objectives and efforts.

Overall, evidence is weak and unclear when it comes to legacy planning, delivery, and outcomes, with many scholars still discussing and advocating for different ways to conceptualize it (Thomson et al., 2019; Byers et al., 2020). This reinforces the complex and multifaceted nature of legacy, which has proved an elusive concept for many MSE hosts and event planners, with limited evidence (especially for Paralympic legacy) to support the successful achievement of legacy outcomes. Coupled with this often-evasive notion of legacy, is the limited connection and focus on (through previous MSEs) the Olympic Movement's Agenda 21 for Sustainable Development. Yet, legacy is seen as an integral part of MSEs' long-term impact, often for social imperatives, providing an opportunity for alignment. As the responsibility of the Olympic Movement to drive positive change has direct synergy with the UN's SDG's, this presents an opportunity (for the UN & IOC) for enhanced collaboration, new partnership models, and stakeholder interaction to create mutually beneficial and long-term change. Given the current global instability and uncertainty, this approach will provide strength and security (through the joint power of the UN and IOC ecosystems, partnerships, and stakeholder groups), especially for the Olympic Movement in a time where the future of live sport events is unclear.

The challenge remains, as to how legacy outcomes can be achieved. If we focus on a critical area of inclusion, through the lens of disability, previous research (Pappous et al., 2011; Brittain and Beacom, 2016; Brown and Pappous, 2018; Annear et al., 2019; Brittain et al., 2020) has demonstrated the challenges and complexity of achieving such Paralympic legacies. This indicates the need for new approaches and ways of thinking to tackle and support legacy production. With digital technologies such as VR and AR becoming more commercialized and publicly accessible (Pan and Hamilton, 2018), this provides greater opportunities for integration and the use of such technologies. VR has been identified as a useful tool to support the achievement of social inclusion and well-being outcomes (Friedman, 2005; Li et al., 2011; Kandaurova and Lee, 2019). Furthermore, previous studies have identified the value of VR when studying social perception and social interaction, as through virtual embodiment, VR's functionality allows examination and control of specific variables (Todorov et al., 2008; Hale and Hamilton, 2016; Pan and Hamilton, 2018). As an example, it would be possible to examine how race and gender interact and influence empathy development (Pan and Hamilton, 2018). Yet, when considering innovation and the use of new technologies such as VR, acceptance and implementation can be challenging, and sport organizations are no exception. We now discuss how innovation has been embraced by sport organizations and acknowledge some challenges associated with the implementation of innovations.

### Innovation in Sport

Joseph Schumpeter coined the term innovation (Hansen and Wakonen, 1997), describing it as an adoption of new technologies or new products (Hartley, 2005; Moore and Hartley, 2008). According to Gjelsvik (2017), innovation could be in the form of a new product, service, production process, administration function, or organizational structure. Innovations can be radical, because of new knowledge, technology, and concepts that have not previously been available (Darsø, 2011). Innovation is fundamental to organization competitiveness and effectiveness but is largely under-researched in the context of non-profit organizations (Wemmer et al., 2016; Byers, 2020). Sport organizations, including federations and local clubs, are a good example of non-profit organizations that create important social value for societies, yet face increasingly turbulent external environmental pressures and internal capacity/resource constraints which place demands on them to seek competitive advantages through innovation and often, collaboration (Wicker and Breuer, 2013). Due to these constraints, some non-profits can be less likely to seek/adopt innovative practices, even though they are in the greatest need of innovation to survive and thrive (Misener and Doherty, 2013).

Innovation is an important tool for organization competency and necessary for survival in the challenging environments in which sport organizations operate (Tjønndal, 2016). It is a tool which challenges leaders of sport organizations to think outside of the box, examine their resourcefulness, and indulge in risk to succeed at the tasks at hand (Hoeber et al., 2015). Non-profit organizations are often limited by their lack of human, financial, and technological resources (Maier et al., 2014; Bach-Mortensen and Montgomery, 2018), and as such these organizations are more inclined to carry out process-based innovations which seek to improve product and/or delivery methods, or innovations focused on aspects that improve organizational structures, learning processes, and environmental adaptation (Edwards-Schachter, 2018). These types of innovations focus on combatting institutional challenges faced by non-profit organizations such as administrative competence, and this careful strategy toward innovation creates a low risk-taking culture in non-profit sport organizations, which could lead to slow progress in goal achievement or falling behind to competitors (Crawford, 2010). The balancing act between increasing risk and growth in these types of organizations calls for a greater need of effective strategy and support from higher management and policy makers.

In their systematic review using a multi-dimensional framework of innovation, Crossan and Apaydin (2010) identified three determinants of innovation within organizations that operate as follows: (1) leadership, found within the upper levels of the organization with capacity to innovate and motivate; (2) managerial levers, responsible for executing organizational mission and goals; and (3) business processes, responsible implementation, project management, and monitoring the process. There are two dimensions of innovation: the process (individual or group level) and the outcome(s). As a process, innovation is driven by internal resources or external market opportunities/pressures. As an outcome, innovation can be a product, service, process, or business model; it can be incremental or radical in magnitude, and it may be firm-specific/ market driven or an industry norm of an administrative or technical type. Gopalakrishnan and Damanpour (1997) distinguish between technical (e.g., production of milk) and administrative (e.g., accounts payable) innovation. It is therefore important to consider the process and barriers to how VR technology may be implemented in sport organizations and the outcomes of implementation, such as social inclusion legacy (Dickson, 2016; Byers, 2020). While the outcomes of VR have been considered in the sport management literature, the process of adopting the technology has received scant attention. We now examine the literature on VR technology and its use in sport.

### Application of Digital Technologies (VR Within and Beyond the Sport Industry)

Within the sport industry, technology is present and ever-changing across a variety of contexts from amateur to professional, on-pitch and off-pitch, and in the areas of spectator experience and consumption (Ratten, 2020). For decades, sport has engaged with new technologies and in turn has experienced change in organization and competition, with Mallen (2019) identifying historical examples such as the development of the wheelchair to exoskeleton technology and golf ball technology. Critically, the sport industry has shown it can embrace change and this evolution has seen innovative, new technologies create opportunities for immersive and engaging experiences. Ratten (2020, p. 1) notes “technology is becoming one of the most important factors driving the international competitiveness of the sport industry.” Even though traditional sport engagement is declining, the use of virtual reality (VR), augmented reality (AR), and artificial intelligence (AI) through interactive media forms such as esports, video games, and streaming platforms, is increasing (Pirker, 2020). These technological approaches and innovations can be utilized and applied to other spheres of the sports industry.

VR has proven a useful innovation on many fronts, from social skills training for people with autism spectrum disorders (Mitchell et al., 2007) to the training of neurosurgeons (Choudhury et al., 2013) and fire fighters (Xu et al., 2014). As early as 2002, although in a neurological rehabilitation context, VR was identified as an application that could help teach disability awareness, by simulating some of the barriers that are experienced by disabled individuals (Sveistrup et al., 2003). Due to the immersive reality that VR technology provides, this offers a mechanism that can be used to shift attitudes and beliefs about disability, which in turn may reduce symbolic ableism. Evidence suggests VR is highly effective in changing attitudes, increasing empathy, and creating greater appreciation for diversity in society, even at the unconscious level through immersive, rich, and isolated experiences (Berson et al., 2018; van Loon et al., 2018). Research on VR in sport has been largely limited to defining the virtual athlete (Jenny et al., 2017), video games and sport consumption behavior (Kim, 2020), analyzing sport performance (Bideau et al., 2010), and rehabilitation (Slobounov et al., 2006), with some limited focus on disability sport (da Cunha et al., 2018).

Beyond sport, VR has been used as a mechanism to support social outcomes, such as well-being and social inclusion across numerous contexts. It is possible to provide lived experiences through VR, which can enhance awareness and lead to attitudinal change (Friedman, 2005; Beadle and Santy, 2008; Li et al., 2011; Kandaurova and Lee, 2019). These applications often align with the broader objective of tackling discrimination and promoting diversity; and researchers have utilized VR to address gender and racial bias and to encourage social inclusion (Beadle and Santy, 2008; Bielen et al., 2018; Lopez et al., 2019). Specifically, studies have found that virtual embodiment of white people in a black virtual body is associated with an immediate decrease in implicit racial bias (Banakou et al., 2016). Interestingly, there have been valuable applications within the education sphere, with Bailenson et al. (2008) noting that virtual environments will transform the learning experience through creating experiences that will alter social interactions and dynamics. VR technology has been utilized by the leading human rights organization UNICEF, to address social issues in education, through the UNICEF Innovation Fund. One such funded project (Imisi 3D) demonstrates how VR can be used to enhance the curriculum, improving the learning experience for Nigerian youth (Virtual Reality in the Classroom, n.d.).

This brief literature review has demonstrated that the concepts of disability, legacy production, and innovation in sport organizations have been extensively explored in isolation, as distinct phenomenon, but their intersections have rarely been considered. Doing so offers a significant opportunity to advance theoretical and practical knowledge to advance the UN's 2030 Agenda for Sustainable Development and social legacy goals of the Olympic/Paralympic Movement. We now turn to a conceptual analysis that elaborates on and draws together understanding of disability, social inclusion, and innovation in sport organizations, that leads to a series of propositions to encourage empirical testing of VR as a tool for social inclusion legacy production.

## Conceptual Framework

Gilson and Goldberg (2015) suggested that good conceptual papers need not propose a new theory at the construct level but should seek to bridge existing theories in interesting ways, link work across disciplines, provide multi-level insights, and/or broaden the scope of thinking. Specifically, we focus this section on applying the model of legacy production presented by Byers et al. (2020) to the Paris 2024 Paralympic legacy. Specific focus is placed on the social inclusion legacy objective for disabled people and the role of technology (VR) and sport organizations to facilitate legacy production. This conceptualization offers a new theoretical perspective, suggesting that sustainable social change can be achieved through MSE legacy, bridging the gap between theory and practice. A discussion follows which outlines four related research propositions.

**Figure 1:**
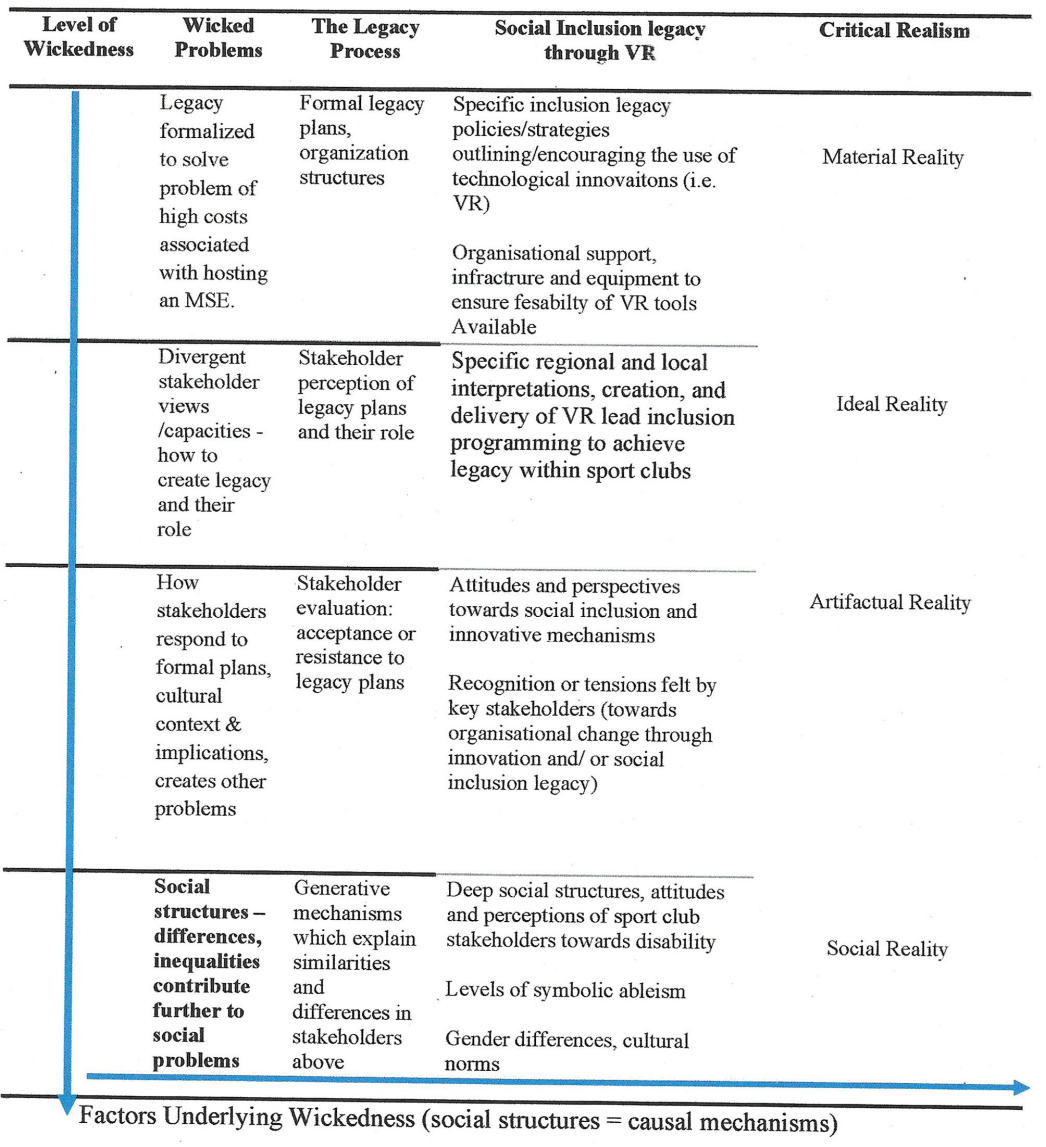
Social inclusion legacy delivery as a “wicked problem”: A critical realist perspective. Factors underlying wickedness (social structures = causal mechanisms). Adapted from Byers et al.'s (2020, p. 177).

Byers et al. (2020) suggested that legacy of MSEs should be explored as a wicked problem which exists through multiple stakeholder lenses and contextual factors, meaning that different solutions/implications to the problem should be considered. To understand innovation management (including barriers to innovation) in non-profit sport organizations and perceptions of disability, a CR approach, as outlined by Byers (2013) and more recently Byers et al. (2020), suggested that complex concepts (e.g., disability and legacy) can be viewed as “wicked problems,” enabling understanding across multiple levels of reality, including the material, ideal, artifactual, and social. Figure 1 presents social inclusion legacy as a wicked problem and demonstrates the role of VR as a potential solution that could facilitate positive social inclusion legacy for disabled people.

Material reality (level 1) consists of formal, tangible policies, procedures, programs, and structures that are in place to manage diversity or define diversity within an organization (e.g., disability sport policy or specific program). This is only a superficial view of reality and it is necessary to examine evidence from the remaining layers of CR to understand how and why the material level exists or is absent from existence (i.e., no provision of disability sport policy or program). A CR perspective of wicked problems provides order and logic to the “wickedness” of social problems through examining different levels of reality and diverse stakeholders' complementary and contradictory assertions. According to CR, reality exists whether we are aware of it or not and so all layers of reality need to be measured/considered by the researcher. Artifactual reality (level 2) focuses on different stakeholder interpretations and meaning of concepts (i.e., perceptions toward disability and innovation). Critically, stakeholders will likely have a diverse, opposing, and complex understanding, as each sports club or federation interprets and determines their position and capabilities to implement VR as an innovative technology within their context.

Ideal reality (level 3) articulates the implications of perspectives in the artifactual layer to reveal if attitudes show that existing practices (material level) are viewed as legitimate or oppressive (i.e., how do individuals respond/act based on their perceptions). This response will drive the stakeholder's acceptance or resistance to such innovative approaches and may be fueled through a positive or negative attitude toward organizational change and technological innovation. The final layer of CR (social layer, level 4) focuses on social structures, such as gender, class, race, or other institutional norms that give rise to and provide a causal explanation for reality and its complementary and contradictory forms. This reality is indicated from the higher levels of reality: material (level 1) or subjective (levels 2 and 3). Especially considering the focus on producing a social inclusion legacy production and the use of VR as a mechanism to overcome/ reduce symbolic ableism, the foundations of the CR methodology are underpinned through the social layer (level 4). It is in this layer that deep-routed social structures, cultures, and values are developed, which create the generative mechanisms which influence the higher layers (levels 2 and 3).

**Figure 2:**
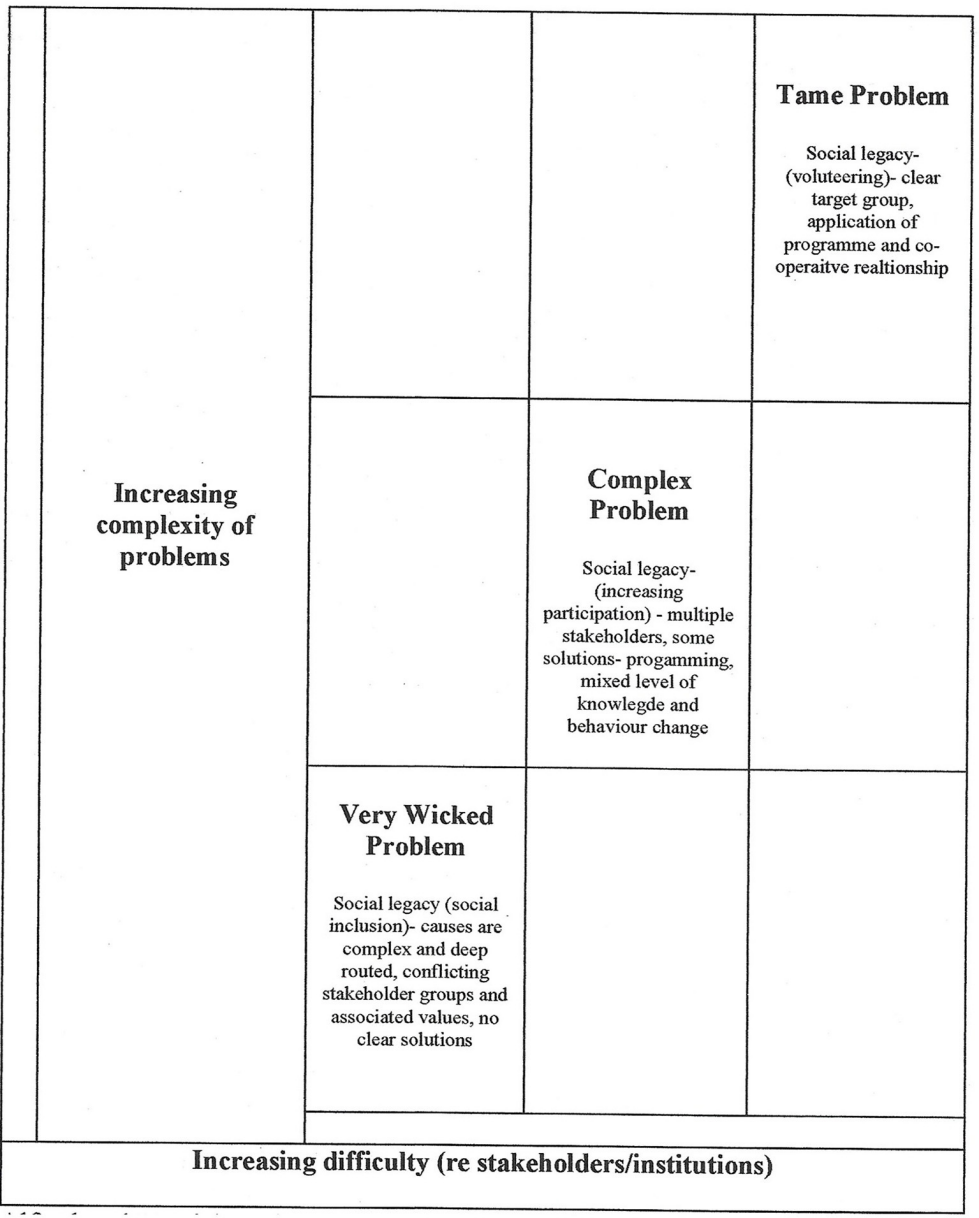
Complexity of wickedness: An illustration. Adapted from Alford and Head (2017) applied to the Paris 2024 social legacy plans.

The wicked problem framework (Alford and Head, 2017) consists of a two-stage model to explore wicked problems, including a vertical axis (level of wickedness) and a horizontal axis (factors underlying the wickedness that illuminate causal mechanisms and explain potential solutions). The level of wickedness is explored though the inherent complexity of the problem, clarity of the problem and potential solutions, and knowledge fragmentation/framing and corresponds well to the levels of reality as illustrated in Figure 1, integrated to illustrate the x and y axes. Figure 2 expands on the application of this framework, specifically in relation to the Paris 2024 legacy plans, and the relative degrees of wickedness. This figure contains illustrative examples (along the diagonal) of different forms of social legacy outlined in the Paris 2024 legacy planning.

Figure 2 elaborates on the “complexity of wickedness” component to illustrate that social legacies such as participation, volunteering, and social inclusion have differences in complexity that are important to legacy production. To understand the Paralympics legacy context, this paper presents legacy as a wicked problem, conceptualized through a critical realist (CR) perspective, to acknowledge the complex layers of reality that comprise legacy production (see Figure 1). Building on Bach-Mortensen and Montgomery (2018) conceptual model which recognized the multiple layers of reality with regards to legacy, this revised model lends itself to exploration of innovation (specifically VR), as a new approach to achieving sustainable legacy outcomes from MSEs related to social inclusion. Critical to this endeavor, sport organizations have been identified as imperative to the delivery of government policy (including legacy delivery and social inclusion), yet research has revealed the significant difficulties these organizations have in meeting the expectations of national and provincial/regional organizations who often hold considerable power to distribute resources (Fahlén et al., 2015). It is therefore important to explore how innovation can be utilized and encouraged in sport organizations to improve effectiveness and to support social impacts such as inclusion for diverse populations. One such innovation that has proven to be highly influential in other contexts (Mitchell et al., 2007; Choudhury et al., 2013; Xu et al., 2014), yet currently has not been considered or explored in legacy research, is VR.

## Discussion: Methodology, Research Propositions, and VR as a Tool for Social Inclusion Legacy

In the previous section, we outlined a conceptual model relevant to the context of Paris 2024, which specifically focused on the potential role of VR as a mechanism to facilitate the production of a positive social inclusion legacy for disabled people. Good conceptual papers also offer propositions as a bridge between validation and usefulness (Weick, 1989) and so this discussion outlines four research propositions which build on the theoretical foundations outlined above. Our propositions have been developed using “CIMO-logic” (context, intervention type, mechanism, outcome) as articulated by Denyer et al. (2008) which develops propositions from a synthesis of existing knowledge (literature). This brings operational clarity to the conceptual model by bridging its strong methodological assumptions (critical realism) and existing theory (wicked problems), incorporating additional theories to specify how under which context (Paris 2024), what interventions (various VR tools) can be implemented (and how) to trigger generative mechanisms (reduced unconscious bias) that lead to specific outcomes (social inclusion for disabled people).

We then explore how a CR methodology can be applied to this context, by outlining relevant implications for future research into Paris 2024 and the achievement of a social inclusion legacy.

### Research Propositions for Social Inclusion Legacy Production Through VR

Taking the Paris 2024 Paralympic legacy goal of social inclusion as a “wicked problem,” we can explore the wickedness of social inclusion and examine the process/barriers to social inclusion to identify the factors contributing to causal mechanisms. Defining social inclusion is wrought with difficulty, it is identified as an important element of well-being for people with physical and intellectual disabilities, yet it can mean different things to different people and occurs through individual, interpersonal, organizational, community, and socio-political pathways (Simplican et al., 2015). These authors also emphasize that inclusion is not just about the availability of opportunity but inherently linked to individual, group, organizational, and societal positivity toward disability. Through a qualitative meta-analysis, Hall (2009) identified six themes defining social inclusion for people with disabilities: being accepted, relationships, involvement in activities, living accommodations, employment, and support systems. These themes also reveal the importance of understanding the influence of attitudes in creating social inclusion. Research on social inclusion in sport for disabled people has revealed that attitudes (including resistance) toward disability is a significant barrier, which requires further research and consideration. This presents a considerable challenge to legacy planning and implementation, as attitudes and attitude change are complex constructs, dynamic in nature, and are dependent on contextual factors.

**Proposition 1:** Unconscious bias (attitudes toward disability and/or social legacy goals) during legacy planning prevent sport clubs from engaging in the legacy planning process, precluding the development of innovative ideas that may facilitate legacy production.

Disability is a form of diversity, and research, policy, and practices on managing diversity in organizations are fragmented and often contradictory in communicating effective practice and the factors which facilitate or hinder policy implementation (Guillaume et al., 2014). Attitudes toward disability can be detrimental to people with disabilities, despite attempts to improve social views and provide equal opportunities for all (Brittain and Beacom, 2016). Brittain et al. (2020) also revealed how ableism can be part of disabled person's attitudes toward their own abilities and so this concept warrants consideration across all stakeholders. Fitzgerald (2018) concludes that barriers to sport participation (attitudinal and structural) are inextricably linked to societal perceptions of people with a disability. Perceptions and interpretation of policy have also been proven strong determinants of sport organization practices across a variety of countries (Donaldson et al., 2012; Fahlén et al., 2015; Jeanes et al., 2019). Understanding perceptions of disability within sport organizations (federations and clubs) is therefore necessary to increase these organization's effectiveness in facilitating social inclusion of persons with a disability.

**Proposition 2:** Level of unconscious bias in sport organizations is related to organization performance and engagement in legacy planning and delivery.

VR has proven highly effective in changing attitudes (Markowitz et al., 2018), increasing empathy (Barbot and Kaufman, 2020; Wiederhold, 2020), and creating greater appreciation for diversity in society (Slater and Sanchez-Vives, 2016), but with these objectives in mind, it has yet to be tested in a sporting/legacy context. Evidence suggests that there are significant attitudinal and structural barriers (Fitzgerald, 2018) to sport participation for persons with a disability, which hampers social inclusion and, in part, explains why participation in sport for this group is significantly less, across many countries, than for persons without a disability (Lauff, 2011; Darcy et al., 2017).

**Proposition 3:** VR sport experiences can increase access and engagement across different sports for disabled people, increasing awareness and motivation to participate in sport.

Several authors have noted that increased organization effectiveness is often a consequence of innovation (Jaskyte, 2004, 2018; Stojcic et al., 2018). Little is known about innovation in non-profit, voluntary organizations (Jaskyte, 2004; Winand et al., 2016). Given Winand's (2016) suggestion that further understanding of the willingness or attitude toward innovation in non-profit sport organizations is needed, the use of VR as a new tool to create a social inclusion legacy presents an ideal opportunity.

**Proposition 4:** Diversity training such as unconscious bias education in sport organizations using VR may increase stakeholder empathy toward disability and increase willingness to engage in the innovation process and use new technologies.

### Methodological Applications

A CR methodology is a multi-level, interdisciplinary approach to reality and knowledge generation that has been embraced in economics, sociology, geography, criminology, history management, and interdisciplinary science studies (Fleetwood and Ackroyd, 2004; Easton, 2010). There are many different methodological approaches which could be explored and considered to test this novel approach to legacy. Yet, given this paper's focus on social inclusion legacy production and the potential use of VR as a mechanism to reduce symbolic ableism, mixed method approaches may provide a fuller understanding (van der Roest et al., 2015) of cultural variations on the perceptions of disability and the role of innovation in sport organizations. CR views mixed methods differently than traditional perspectives of the role of quantitative methods.

Specifically, one methodological approach that could be considered involves focus groups, with mixed stakeholder groups to understand and explore perceptions and attitudes toward disability and innovation. By using the CR layers of reality as a guiding framework, this would lead to rich discussion and insight into the constructs that support or hamper legacy production. By understanding the causal mechanisms (social layer), it is possible to understand how they have informed certain attitudes (artifactual layer), behaviors (ideal layer), and tangible strategies/policies (material layer) of said stakeholders. This could then be triangulated with a quantitative component which would focus on assessing unconscious bias (Friedman and Awsumb, 2019), to enhance validity, examine the extent of ableism in sport organizations, and thereby narrow the parameters of the research focus, as per the unique application of mixed methods in CR methodology (Zachariadis and Barrett, 2013). A specific quantitative scale that could be utilized in a survey for example is the Symbolic Ableism Scale (SAS). The SAS is a validated tool that measures a key aspect of capacity (unconscious bias) in sport organizations that is often overlooked but through the CR ontology. Cross-cultural research in this regard would be beneficial to understand how this approach may serve the Olympic Movement and legacy of the Paralympic Games. This is one possible methodological design that could be considered to test this conceptual articulation of legacy, yet we are not suggesting that this is the only means, for example a quasi-experimental design could be employed, and VR diversity training could be offered to sports organizations, alongside a pre- and post-test of symbolic ableism to evaluate and explore the effects of VR technology on unconscious biases.

VR is an innovative and untested tool in this context, with the potential to change conscious and unconscious bias toward disability and other social structures such as gender (Banakou et al., 2016; Slater and Sanchez-Vives, 2016). Critically, it is important to recognize that innovation is not a new phenomenon, yet it has not been explored in relation to legacy. Legacy has been identified as a challenging, contested, and fluid concept, which is difficult to define and inclusive of deep social structures which aid or hinder legacy delivery (Brownill et al., 2013; Byers et al., 2020). Therefore, this paper proposes that due to the complexity of legacy production, which has resulted in previous MSEs struggling to deliver their legacy objectives, innovation is needed. Specifically, an approach that moves beyond tangible policies, leveraging strategies or narrow, single disciplinary conceptual perspectives (e.g., Thomson et al., 2020), and examines the deeper social structures and attitudes and contextual parameters of disability and innovation, through utilizing Bach-Mortensen and Montgomery (2018) model, can help determine the degree of a legacy's wickedness. From that, innovation might provide a mechanism through which disruption and significant change (i.e., social inclusion) can be achieved.

## Conclusions

This paper proposes the use of dynamic, technologically-fueled innovation to aid in the development and delivery of legacy, particularly a social inclusion legacy for the Paris 2024 Olympic/Paralympic Games. VR has been utilized and thrived in other contexts within the sport, entertainment, and media industries, and therefore it is possible for new applications to be realized. As decades of legacy research has identified, legacy is not only a complex, ever-changing concept, but successful legacy production has proven obscure. Therefore, we suggest innovation should be a core component of legacy planning for future MSE hosts to deal with its “wickedness.” However, it must be noted this will require a culture shift in not only how legacy is understood and articulated, but critically, an engagement with new and novel approaches to aid legacy production. Having an open, dynamic approach to legacy production and planning will allow new approaches such as VR to be considered. This could facilitate more collaborative partnerships to achieve broader global social objectives (such as inclusion, equality, and sustainability) through the power of MSEs. As for the long-term sustainability, growth, and interest/popularity of the Olympic Movement, new approaches and processes need to be adopted to ensure the relevance and viability of such a historic entity. This may also open new markets (of high relevance for the IOC, who have ever decreasing viewer numbers/aging audiences), especially with younger audiences, who are highly engaged and part of a digital society.

The application of innovative technologies such as VR to achieve a better future for all (in line with both the legacy objectives for Paris 2024 and SDGs), requires stronger alignment and collaboration between multiple industries and partners, such as relevant sport for development, MSE, and digital technology and VR stakeholders. Furthermore, when reflecting on the micro level and the delivery of VR applications through sports clubs, this is going to require acknowledgment, willingness, and ability to use and apply new technological innovations and products to aid the achievement of such complex global challenges, such as inclusion through the Olympic Movement. This will itself be a challenge as previous research has noted that despite the advantages of sport technology, some sport organizations are reluctant to adopt technology because of a wish to continue the status quo (Mallen, 2019).

Further research on innovation in sport organizations, including governing bodies and clubs, is needed to understand their perceptions of innovation and the challenges of implementing innovations such as VR. Critically, Manzoor and Vimarlund (2018) have noted that there has been limited research that has examined how technological applications can be used to facilitate social inclusion for individuals with disabilities, supporting the need for future investigation. Therefore, we propose multiple research propositions that need to be empirically examined and investigated further, across numerous contexts to understand the “wickedness” of legacy production. Research to establish the extent of symbolic ableism in sport organizations would also help understand the problem in more depth and breadth, if a global sample could be obtained.

## Author Contributions

TB, EH, and FM equal contributors to conceptual construction of paper. PL and DH student contributors to literature review and referencing. All authors contributed to the article and approved the submitted version.

## Conflict of Interest

The authors declare that the research was conducted in the absence of any commercial or financial relationships that could be construed as a potential conflict of interest.
